# Travel Distance Between Participants in US Telemedicine Sessions With Estimates of Emissions Savings: Observational Study

**DOI:** 10.2196/53437

**Published:** 2024-05-15

**Authors:** Mollie R Cummins, Sukrut Shishupal, Bob Wong, Neng Wan, Jiuying Han, Jace D Johnny, Amy Mhatre-Owens, Ramkiran Gouripeddi, Julia Ivanova, Triton Ong, Hiral Soni, Janelle Barrera, Hattie Wilczewski, Brandon M Welch, Brian E Bunnell

**Affiliations:** 1 College of Nursing University of Utah Salt Lake City, UT United States; 2 Spencer Fox Eccles School of Medicine Department of Biomedical Informatics University of Utah Salt Lake City, UT United States; 3 Doxy.me Inc Charleston, SC United States; 4 Department of Geography University of Utah Salt Lake City, UT United States; 5 Department of Psychiatry and Behavioral Neurosciences Morsani College of Medicine University of South Florida Salt Lake City, UT United States; 6 Medical University of South Carolina Charleston, SC United States

**Keywords:** air pollution, environmental health, telemedicine, greenhouse gases, clinical research informatics, informatics, data science, telehealth, eHealth, travel, air quality, pollutant, pollution, polluted, environment, environmental, greenhouse gas, emissions, retrospective, observational, United States, USA, North America, North American, cost, costs, economic, economics, saving, savings, finance, financial, finances, CO_2_, carbon dioxide, carbon footprint

## Abstract

**Background:**

Digital health and telemedicine are potentially important strategies to decrease health care’s environmental impact and contribution to climate change by reducing transportation-related air pollution and greenhouse gas emissions. However, we currently lack robust national estimates of emissions savings attributable to telemedicine.

**Objective:**

This study aimed to (1) determine the travel distance between participants in US telemedicine sessions and (2) estimate the net reduction in carbon dioxide (CO_2_) emissions attributable to telemedicine in the United States, based on national observational data describing the geographical characteristics of telemedicine session participants.

**Methods:**

We conducted a retrospective observational study of telemedicine sessions in the United States between January 1, 2022, and February 21, 2023, on the doxy.me platform. Using Google Distance Matrix, we determined the median travel distance between participating providers and patients for a proportional sample of sessions. Further, based on the best available public data, we estimated the total annual emissions costs and savings attributable to telemedicine in the United States.

**Results:**

The median round trip travel distance between patients and providers was 49 (IQR 21-145) miles. The median CO_2_ emissions savings per telemedicine session was 20 (IQR 8-59) kg CO_2_). Accounting for the energy costs of telemedicine and US transportation patterns, among other factors, we estimate that the use of telemedicine in the United States during the years 2021-2022 resulted in approximate annual CO_2_ emissions savings of 1,443,800 metric tons.

**Conclusions:**

These estimates of travel distance and telemedicine-associated CO_2_ emissions costs and savings, based on national data, indicate that telemedicine may be an important strategy in reducing the health care sector’s carbon footprint.

## Introduction

Diminished air quality, marked by high levels of pollutants and particulate matter, has been associated with varied cardiovascular, respiratory, and other health issues and premature mortality [1-12]. Fossil fuel–based transportation directly causes air pollution by releasing particulate matter, nitrogen oxides, and volatile organic compounds. Additionally, the emission of greenhouse gases (GHGs), including carbon dioxide (CO_2_) from fossil fuel consumption (including transportation-related consumption), contributes profoundly to climate change [13]. The heat-trapping properties of CO_2_ and other GHGs are considered primary drivers of global warming [14]. With climate change, we anticipate that air quality and human health will be further diminished by increased levels of ground-level ozone, particulate matter due to wildfires, and airborne allergens [13]. Beyond its effects on air quality, climate change is associated with varied additional health problems resulting in morbidity and mortality, ranging from heat-related illness to infectious disease transmission and food insecurity [15]. During the first year of the COVID-19 pandemic, we observed sharp decreases in atmospheric CO_2_ worldwide, attributed to decreases in transportation and other CO_2_-emitting human activities in compliance with public health orders [16-18]. However, atmospheric CO_2_ has since increased and is forecasted to rise with fossil fuel consumption in the coming years, and there is a critical, global need to reduce emissions and mitigate impacts on human health [18,19].

Ironically, hospitals and health care organizations are major contributors to CO_2_ emissions. These emissions relate to both direct and indirect energy expenditures of health care facilities, many of which operate 24 hours per day, 7 days per week. Emissions also result from the transportation of health care providers and other employees who provide services, and the transportation of patients and other visitors traveling to facilities. Most health care emissions result from the supply chain, which is dependent upon fossil fuels and includes the production, transportation, and operation of equipment, devices, and material supplies [20]. In a 2016 EIO-LCA (economic input-output life cycle assessment) modeling analysis, Eckelman and Sherman [21] estimated that 10% of US CO_2_ emissions were attributable to the health care sector in 2013, prompting a re-examination of health care delivery practices in light of the environmental impact. In a 2020 update to the original analysis, the authors analyzed more recent data and incorporated additional state-level analyses of access and quality [22]. They determined that the US national health care GHG emissions increased by 6% from 2010 to 2018 and that electricity is the largest contributor to GHG emissions from the health care sector [22]. Health care pollution is a growing global concern. It is estimated that the health care sector is responsible for 4.4% of global GHG emissions and the United States appears particularly culpable [20]. In an international comparison of health carbon footprints, defined as global supply chain CO_2_ emissions related to health care expenditures and investments, the United States was the second largest emitter of health care–related CO_2_ emissions [23].

Telemedicine is a potentially important strategy for mitigating health care related GHG emissions, and numerous studies have examined the GHG emissions attributable to telemedicine. A 2022 systematic review of 31 studies conducted between 2000 and 2021 by Donald and Irukulla [24] found that telemedicine is associated with substantial CO_2_ emission savings. However, previous US studies that examined telemedicine-associated CO_2_ emissions savings were conducted entirely in local or regional settings [24]. For example, a 2021 study by Jiang et al [25] examined travel-related emissions savings in a convenience sample of 100 veterans receiving teleoncology care at a single site. They estimated that there was a savings of 35.5 metric tons of CO_2_ with 560 sessions. In a recent larger study conducted at Stanford Health Care in California, researchers estimated a savings of 17,000 metric tons in 2021 due to telemedicine use [26].

However, specific estimates of CO_2_ emissions savings from prior research vary widely, as they are based upon varied assumptions, regions, and medical specialties. Among US studies, estimates of the CO_2_ emissions savings per telemedicine session range from 11.2 (vascular surgery, Michigan) to 893 kg CO_2_ (otorhinolaryngology head and neck surgery, New Mexico) [24,27,28]. Additionally, most estimates are based on fairly small regional samples with under 500 participants. In the Donald and Irukulla [24] review, only 2 US studies, 1 conducted in California and 1 conducted in Utah, had more than 1000 participants [29,30]. Consequently, the overall extent of telemedicine’s contribution to CO_2_ emissions savings in the United States is unknown.

Health care pollution is a critical concern, and we must consider environmental impacts including GHG emissions when designing health care programs, services, and facilities to avoid unintended adverse consequences on human health. Digital health and telemedicine are potentially important strategies to decrease the health care sector’s adverse environmental health impacts. However, we currently lack robust national estimates of emissions savings attributable to telemedicine that would enable the quantification of telemedicine’s emissions savings in modeling efforts. This study aimed to (1) determine the travel distance between participants in US telemedicine sessions and (2) estimate the net reduction in CO_2_ emissions attributable to telemedicine in the United States, based on national observational data describing the geographical characteristics of telemedicine session participants.

## Methods

### Study Design

We conducted a retrospective observational study of telemedicine sessions in the United States between January 1, 2022, and February 21, 2023, on the doxy.me platform. We calculated the approximate travel distance between providers and patients for a proportional sample of sessions, including estimated emissions savings and expenditures. Further, based on the best available public data, we calculated the emissions savings attributable to telemedicine in the United States.

### Ethical Considerations

The data used in this study were determined to be deidentified, as per the Health Insurance Portability and Accountability Act (HIPAA) of 1996, using the expert determination method. All study procedures were reviewed by the University of Utah Institutional Review Board and determined to be nonhuman subjects research.

### Doxy.me Session Data

Doxy.me is a commercial telemedicine platform commonly used by individual providers, clinics, and health care organizations [31]. Based on third-party survey research and other estimates, doxy.me is used in 8%-30% of telemedicine sessions in the United States daily [32-34]. A HIPAA-compliant platform, it does not store patient names, addresses, or medical information. For operational purposes, doxy.me stores the IP addresses of session participants and limited account information about providers. For this study, we defined a telemedicine session as any session between 1 patient and 1 provider, with a duration between 5 and 120 minutes. Doxy.me assigned approximate locations (geospatial coordinates) to the telemedicine sessions based on IP addresses using a free IP geolocation service, ipstack (iPstack API).

### Sampling and Time Frame

We analyzed a proportional sample of 79,904 sessions, drawn from a randomized sample of 8,000,000 doxy.me telemedicine sessions that occurred in the United States, between 2 participants (1 patient and 1 provider), between January 1, 2022, and February 21, 2023, meeting inclusion criteria and less than 400 miles geodesic distance. The strata used for proportions were call region (Northeast, Southeast, West, Midwest, and Southwest); session length (5-30 minutes, or greater than 30 minutes); day of the week (Monday through Friday or weekend); the hour of the day (1 PM through 11 PM Coordinated Universal Time, or all other hours). Then, we calculated travel distance and time using the Google Distance Matrix Application Programming Interface for car travel [35].

### Inclusion Criteria

Telemedicine sessions occurring between dyads (1 provider and 1 patient), using any device, between January 1, 2022, and February 21, 2023.

### Exclusion Criteria

We excluded calls of less than 5 minutes, as calls of this duration generally do not represent actual clinical encounters. Rather, they are attributable to changes in equipment or devices or a need to adjust settings. We also excluded group sessions and sessions longer than 2 hours, which likely represent the use of doxy.me for remote monitoring. We excluded sessions with >400 miles geodesic distance to mitigate bias related to extreme distances and based on the conservative assumption that most patients receiving care from a highly distant health care provider, should they need to see that provider in person, would seek alternative local care or not seek care at all.

### Analysis

For the proportional sample of 79,904 sessions, we first geocoded the location of health care seekers and providers based on the coordinates derived from IP addresses, then calculated car travel time and distance using the Google Distance Matrix Application Programming Interface [35]. We calculated descriptive statistics and box plots to describe session characteristics, including duration, specialty, patient travel time, and travel distance (round trip). Then we adjusted the travel distance for a more realistic approximation of travel savings due to telemedicine. We multiplied by 0.848, based on the assumption that approximately 84.8% of travel was car travel, consistent with American Community Survey data on adult commuting patterns in the United States [36].

### CO_2_ Emission Savings

We calculated the tailpipe CO_2_ emission savings associated with the telemedicine sessions by multiplying the total adjusted travel distance in miles by 404 grams, which is the 2022 US Environmental Protection Agency estimate of CO_2_ emissions per mile for an average passenger vehicle, assuming an average fuel economy of 22.0 miles per gallon [37].

### CO_2_ Expenditure

To approximate the CO_2_ expenditure associated with each telemedicine session, we first calculated the energy expenditure associated with videoconferencing using the estimates and methods described by Blenkinsop and colleagues [38] and Mytton [39], corresponding to the use of 720p high-definition video by 2 participants. Following that approach, we calculated the total amount of data transferred during each session using a rate of 0.036 GB per minute [38]. We assumed an electricity use of 0.015 kWh/GB, based on fixed-line energy transmission estimates [40]. Using the US Environmental Protection Agency conversion rate of 4.33 × 10^–4^ metric tons CO_2_/kWh, we estimated the CO_2_ emissions expenditure in metric tons. Then, we subtracted the CO_2_ expenditure from CO_2_ savings to calculate the net CO_2_ savings per session.

No publicly available data describes the total number of telemedicine sessions that took place in the United States during the dates of this study. To obtain an approximate estimate of annual US telemedicine sessions, we used publicly reported data on the number of telemedicine sessions reimbursed via the US Medicare and Medicaid programs. Centers for Medicare & Medicaid Services (CMS) spending accounted for 38% of National Health Expenditures in 2021. CMS reimbursed for 27,691,878 telemedicine sessions during 1 year, from March 1, 2020, to February 28, 2021 [41]. While expenditures do not translate directly to the volume of health care delivery or the number of telemedicine sessions that take place, we used it as a best-available approximate of the overall volume of health care delivered using telemedicine. Based upon these figures, we estimate that CMS-reimbursed telemedicine sessions represented 38% of a total of 72,873,363 telemedicine sessions that occurred in the United States in 2021, and we base our estimate of CO_2_ emissions savings on this number of sessions and the postpandemic policy environment of 2021 and 2022.

### Representativeness of Doxy.me Session Data

We used multiple data sources describing national telemedicine use patterns to assess the representativeness of doxy.me data for national patterns of use [41-44]. We calculated descriptive statistics for 2 points of comparability, the regional distribution of sessions and the specialty of providers, and compared the observed distributions with that reported nationally for Medicare claims. See Multimedia Appendix 1 for a detailed comparison.

### Geographic Distribution of CO_2_ Emissions Savings

Using ArcGIS, we created 2 maps that visualize CO_2_ emissions savings at the zip code level. The first map shows the overall pattern of CO_2_ savings. The second map shows the CO_2_ savings per session.

## Results

### Overview

A random sample of 8,000,000 telemedicine sessions, hosted by doxy.me, between January 1, 2022, and February 21, 2023, were examined. Of the sample, 6,231,614 sessions met our inclusion and exclusion criteria. After proportional sampling, the analytic data set consisted of 79,904 sessions.

### Session Characteristics

**Figure 1:**
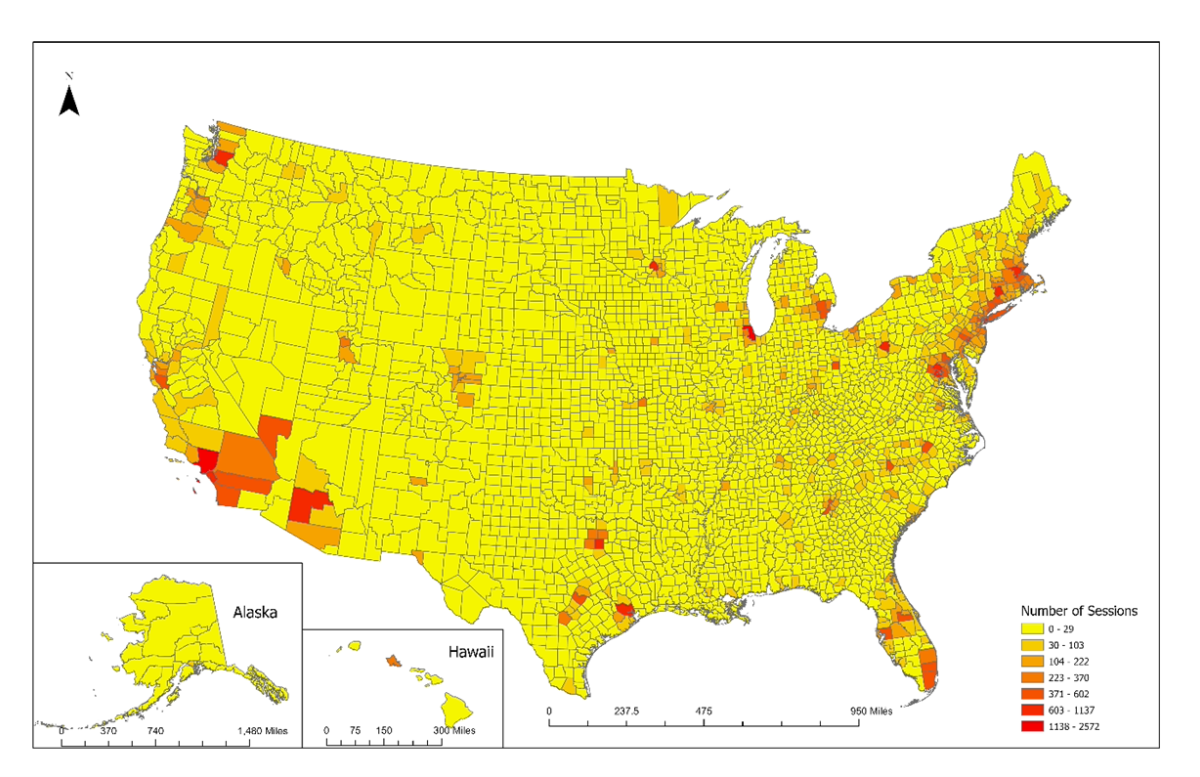
Geographical distribution of telemedicine sessions and total number of sessions by county.

**Figure 2:**
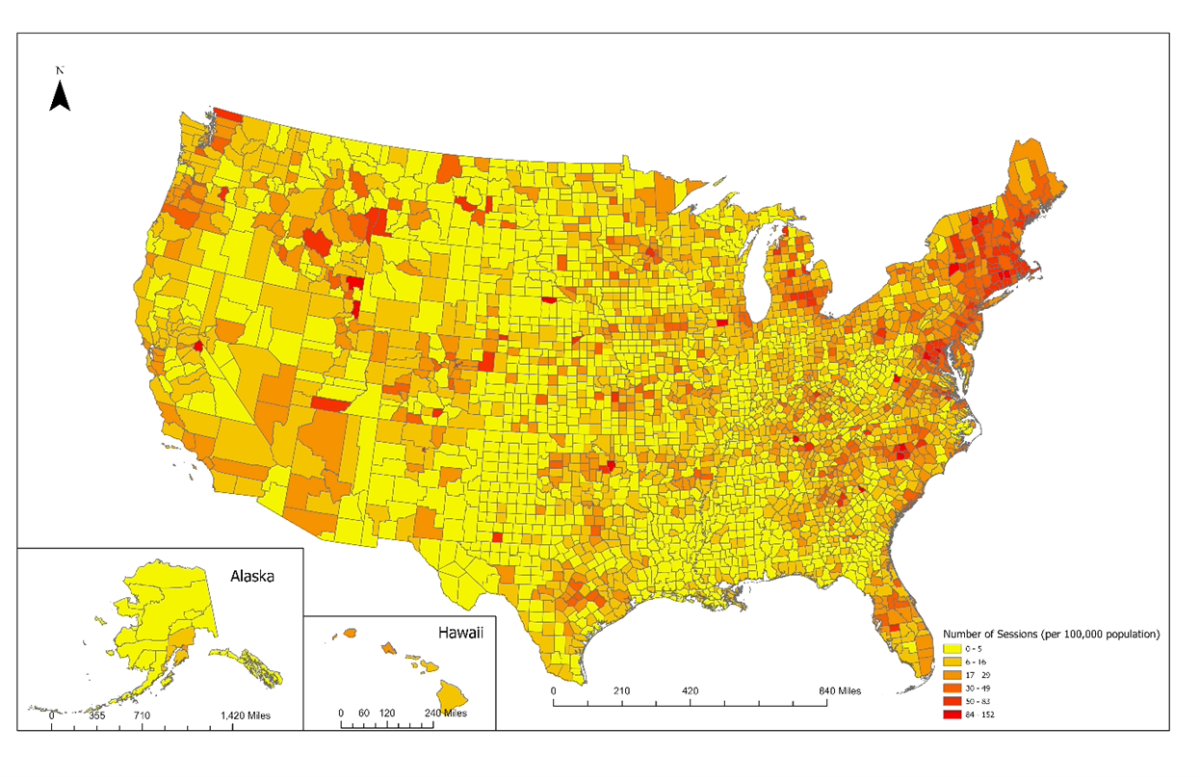
Geographical distribution of telemedicine sessions and number of sessions per 100,000 population by county.

The duration of sessions was bimodal in distribution. Of the 79,904 telemedicine sessions, 35,204 (44.06%) were 5-30 minutes in length, and 44,700 (55.94%) were >30-120 minutes in length. The Northeast region had the most sessions 27,311 (34.18%), followed by the Southeast 16,318 (20.42%), West 15,179 (18.99%), Midwest 13,788 (17.26%), and finally Southwest 7308 (9.15%). Tuesday, Wednesday, and Thursday were most frequent: 18,134 (22.69%), 17,751 (22.22%), and 17,052 (21.34%), respectively. From 2 PM-5 PM Eastern (11 AM-2 PM Pacific) were the most common hours, each at approximately 8150 sessions (10.19%). The geographic distribution of the sessions by region is described in Table 1 and depicted in Figures 1 and 2. Comparisons of the doxy.me data to reference data sources, by state and by specialty are provided in Multimedia Appendix 1.

**Table 1:** Session characteristics include duration and geographic distribution.

Characteristics			Full data set (N=6,231,614), n (%)		Proportional sample (n=79,904), n (%)
**Duration (min)**					
	5-30	2,747,610 (44.06)		35,204 (44.06)	
	30-120	3,484,004 (56.94)		44,700 (55.94)	
**Geographical location (by region)**					
	Northeast	2,125,693 (34.18)		27,311 (3.18)	
	Southeast	1,272,704 (20.42)		16,318 (20.42)	
	West	1,183,814 (18,99)		15,179 (18.99)	
	Midwest	1,076,509 (17.26)		13,788 (17.26)	
	Southwest	572,884 (9.15)		7308 (9.15)	
	Missing	10 (0)		0 (0)	

### Travel Distance

**Figure 3:**
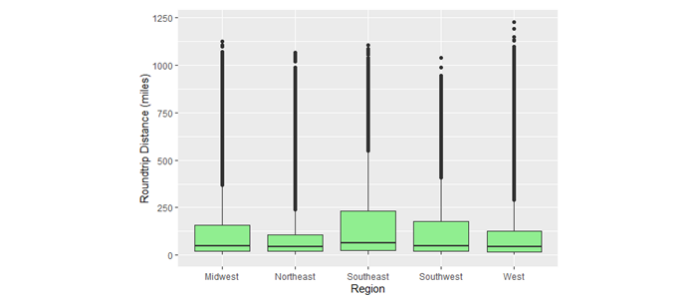
Boxplots of round trip travel distance (miles) by US region for proportional sample (N=79,904).

Travel distance was not normally distributed. Overall, the median round trip distance was 49 (IQR 21-15) miles; the mean round trip travel distance was 134 (SD 196) miles. Descriptive statistics for round trip travel distance (in miles) grouped by day of week, region, and telehealth session length are presented in Table 2. Weekends show the highest round trip distance (mean 150, SD 210, and median 52, IQR 23-182). The Southeast region showed the highest distance of travel (mean 167, SD 214, and median 63, IQR 25-234). Telemedicine sessions of 5-30 minutes duration showed the highest travel distance (mean 144, SD 204, and median 54, IQR 23-166). Figure 3 shows box plots of round trip travel distance, by region. The box plots illustrate regional variation, the skewed distribution of distance across all regions, and the presence of high-distance outliers.

**Table 2:** Round trip travel distance (miles) for proportional sample (N=79,904).

Characteristics		Sample, n	Mean (SD)	Median (IQR)	Minimum to maximum
**Day of week**					
	Monday	13,777	131 (194)	49 (20-141)	0-1228
	Tuesday	18,134	132 (195)	49 (20-144)	0-1191
	Wednesday	17,751	135 (198)	49 (20-147)	0-1130
	Thursday	17,052	133 (195)	49 (21-143)	0-1098
	Friday	10,711	136 (198)	50 (22-149)	0-1065
	Saturday and Sunday	2479	150 (210)	52 (23-182)	0-1065
**Region**					
	Midwest	13,788	143 (209)	48 (10-159)	0-1127
	Northeast	27,311	106 (157)	46 (20-109)	0-1065
	Southeast	16,318	167 (214)	63 (25-234)	0-1104
	Southwest	7308	145 (205)	49 (22-177)	0-1040
	West	15,179	136 (217)	44 (18-128)	0-1228
**Session length**					
	30-120 min	44,700	126 (190)	46 (19-130)	0-1228
	5-30 min	35,204	144 (204)	54 (22-166)	0-1130

### Emissions Savings and Expenditures

The emissions savings and expenditures are detailed in Table 3. The mean CO_2_ savings per telemedicine session was 54,077 (SD 79,362) g and the median was 19,812 (IQR 8322-58,772) g. The mean CO_2_ savings per minute of telemedicine care was 2896 (SD 6258) g, and the median was 723 (IQR 239-2597) g. For the 6,231,614 doxy.me telemedicine sessions, we estimate a savings of 123,461 metric tons of CO_2_ (calculated with median). Further, we estimate that nationally, the use of telemedicine in the United States during the years 2021-2022 resulted in approximate annual CO_2_ emissions savings of 1,443,800 metric tons (calculated with median). Multimedia Appendix 2 contains a map showing the geographical distribution of the CO_2_ emissions savings per telemedicine session.

**Table 3:** Travel and emissions calculations per session for proportional sample (N=79,904).

Characteristics	Mean (SD)	Median (IQR)	Minimum to maximum
Travel time, round trip, automobile^a^ (h)	2.38 (3.01)	1.15 (0.64-2.66)	0.00 to 22.94
Travel distance, round trip, automobile^a^ (miles)	133.87 (196.44)	49.05 (20.62-145.49)	0.00 to 1227.55
Transportation-related CO_2_ emissions savings (g)	54,085.06 (79,362.04)	19,818.13 (8331.32-58,776.59)	0.00 to 495,928.64
Duration (min)	34.53 (20.03)	36.73 (14.48-51.73)	5.00 to 119.83
CO_2_ emissions equivalent of telemedicine energy use (g)	8.07 (4.68)	8.59 (3.39-12.09)	1.17 to 28.02
Net emissions savings per telemedicine session (g)	54,076.99 (79,362.26)	19,812.45 (8322.11-58,772.37)	–27.89 to 495,921.13
Net emissions savings per minute of session length (g)	2896.17 (6258.47)	722.99 (238.65-2596.56)	–0.23 to 79,230.29

^a^Calculated using Google Distance Matrix Application Programming Interface.

## Discussion

### Principal Findings

We used national, observational data to determine the travel distance between patients and providers participating in telemedicine sessions. Further, we used our findings to generate national estimates of CO_2_ emissions costs and savings. Under the relatively stable relevant policy conditions of the years 2021-2022, we estimate the annual emissions savings associated with US telemedicine as 1,462,932 metric tons or nearly 1.5 million metric tons. This is equivalent to the CO_2_ emissions from approximately 3.4 million barrels of oil or 165 million gallons of gasoline [45]. This estimate is unique in that it was based upon a large national set of observational data, calculation of travel distance using Google Distance Matrix, and a set of conservative assumptions related to telemedicine connections and behavior. Further, we estimated emissions savings per telemedicine session and per minute of telemedicine care delivery, potentially useful estimates for modeling the environmental impact of health care programs and services. Previously published estimates of emissions savings were based on regional or local telemedicine use and are not directly comparable. Here, we generated estimates based on a large national sample of US telemedicine sessions conducted in varied geographic areas by health care providers of varied specialties.

We used round trip travel distance determined using Google Distance Matrix rather than geodesic distance as the basis for emissions savings estimates. With geodesic distance ≤400, travel distance by car still exceeded 1000 miles in some rare cases, particularly in the West, but was typically much lower. We found that travel distance varied according to region and day of the week. On weekends, there were fewer telemedicine sessions, but the travel distance between providers and patients was greater. This may reflect weekend telemedicine staffing patterns that use more distant health care providers, or the use of telemedicine services based in more distant geographical areas when local, in-person health care services are closed over a weekend. Additionally, we observed some regional variation in the distribution of travel distance. We observed the highest median travel distance between patients and providers in the southeastern United States, and the lowest median distance in the West. Additionally, shorter sessions were characterized by greater distances, which could reflect decisions to manage brief encounters such as follow-up visits by telemedicine when patients are particularly distant from the health care provider.

We found a median round trip travel distance by automobile of 49 (IRQ 21-145) miles, based on the approximate locations of patients and providers participating in telemedicine sessions. This estimate is far lower than the travel distance savings found in larger prepandemic research studies [24]. For example, Thota et al [29] found a mean round trip distance savings of 332 miles per encounter for telemedicine cancer care in rural Utah. A prepandemic study of telemedicine consultations in the UC Davis system found a mean round trip distance savings of 278 miles [30]. A larger and more recent pandemic-era study of telemedicine in 5 UC health systems showed a substantially lower round trip travel distance savings of 17.6 miles, likely reflecting widespread pandemic-era telemedicine adoption and increased use of telemedicine in urban areas of the United States [46]. Our results align most closely with those of Sharma and colleagues [46], consistent with the similar timeframe and pandemic-era telemedicine use patterns. Our results likely differ because we used an entirely different approach to calculating travel distance; we used Google Distance Matrix to calculate automobile travel distance based on observational data describing the approximate actual location of patients and providers during telemedicine sessions, rather than geodesic (point-to-point) distance and historical street address data as in most previous studies. Further, our analysis was based on national data that reflects greater geographical diversity and diversity in patterns of telemedicine use.

To slow global warming and climate change, we must reduce CO_2_ emissions in the health care sector. Though we estimate a substantial net savings of CO_2_ emissions through telemedicine, these savings are equivalent to merely 0.02% of the total US CO_2_ emissions in 2021 (6340.2 million metric tons) [47]. Telemedicine is clearly important in reducing GHG but must be combined with other innovative programs, services, and changes in behavior within and beyond the health care sector. An increasing number of health care organizations in the United States have committed to reducing their carbon footprint. For example, Kaiser Permanente achieved carbon neutrality in 2020, and the University of California San Francisco is working toward carbon neutrality by 2025 [48]. Given our findings, the environmental impact of telemedicine should be duly considered in designing programs and services, as part of the effort to reduce health care’s carbon footprint.

### Limitations and Considerations

The estimates in this study depend upon a series of assumptions related to energy-consuming human behaviors. However, many of the assumptions are conservative and, if erroneous, would underestimate rather than overestimate CO_2_ emissions savings. For example, we used adult commuting patterns to determine the percentage of travel occurring by car. However, patients who use alternative transportation to work (eg, public transportation, biking, and walking) may still travel by car to health care appointments. However, the use of cars for travel to health care appointments may differ for children, the retired, old adults, or disabled persons.

Similarly, we assumed patients would not travel to in-person appointments in place of telemedicine when the geodesic distance exceeds 400 miles and excluded sessions with a geodesic distance >400 from the analysis. Given this distance, if telemedicine was not an option, we reason that patients would have sought alternative local care or missed care. In reality, a certain proportion of these patients may travel unusually long distances, especially those seeking specialty care (eg, care at a cancer center) and patients in frontier areas. However, we decided to exclude high-distance telemedicine sessions to avoid inflated estimates.

Another critical assumption is that doxy.me data are nationally representative of patients and providers participating in telemedicine. We compared the geographic distribution of doxy.me session data to that reported for Medicare fee-for-service payments in Grace [44] by state. We found them to be highly similar (Multimedia Appendix 1). States with higher dense populations, such as California, New York, and Texas, are consistent in higher telemedicine session use in both sources. In contrast, more rural states like Wyoming have consistently low use (Multimedia Appendix 1). The overall market share of the doxy.me platform has been estimated by third parties as between 7% and 30% of US telemedicine [32-34,49-51].

For doxy.me specialty data, we were limited to the data available through deterministic linkage of doxy.me provider accounts to NPPES (National Plan and Provider Enumeration System), which was available for only 43.55% of the sessions. To gain insight into whether the doxy.me data are nationally representative in terms of specialty, we compared the distribution of specialty in the doxy.me data to that of the Medicare fee-for-payment data described by Grace [44]. There were marked differences in the distribution of specialties, which may or may not be attributable to quality issues in the linked specialty data (Multimedia Appendix 1). However, mental health services, psychiatry, and social work were consistent and frequent specialties in both doxy.me and the Medicare data. The types of telemedicine supported by the doxy.me telemedicine platform may differ somewhat from those of other platform providers. Doxy.me has a firm market share among mental health providers, offering integrated digital health functionality for mental health care. Thus, the sample that we analyzed may overrepresent mental health services and underrepresent other health care specialties (Adhere.ly; Adhere.ly, LLC). Given the large amount of missing data for specialty and substantial differences compared to Medicare claims data, we did not estimate telemedicine emissions savings by provider specialty.

The total number of telemedicine sessions that occur in the United States during a given year is unknown. We based our estimate on actual CMS reimbursement for telemedicine during 2021 and the proportion of US health care expenditures funded by CMS. Both 2021 and 2022 were years during the federal health emergency declaration related to the COVID-19 pandemic. Federal and state policies related to reimbursement and licensing can substantially affect telemedicine use patterns. However, policy was relatively stable during the years 2021-2022.

In future work, we plan to update and refine these estimates based on observational data describing real-world telemedicine connection characteristics and the type of devices used to connect, which affect the energy costs associated with telemedicine. We also plan to incorporate energy costs associated with standard, in-person care delivery including built infrastructure and personnel transportation. More robust specialty data would enable additional insights into potential variations in CO_2_ emissions costs and savings across different types of health care. Additionally, future efforts could refine model assumptions based on observed health care use behavior patterns and regional differences in health care service delivery, as the body of evidence describing telemedicine use grows.

### Conclusions

US health care is under increasing scrutiny for contributing to air pollution, climate change, and related adverse health outcomes. To duly consider environmental impacts in decision-making related to health care facilities, programs, and services, we must be able to quantify and compare the environmental effects of varied approaches to health care delivery. Here, we determined the travel distance between participants in US telemedicine sessions and, on that basis, estimated the associated annual CO_2_ emissions savings for US telemedicine in 2021-2022 as nearly 1.5 million tons. Further, we estimated emissions savings per telemedicine session, and per minute of telemedicine care delivery based on a large national sample of telemedicine sessions. In doing so, we adopted conservative assumptions related to patient behavior and transportation and considered both energy savings and expenditures of telemedicine. The results indicate that telemedicine is an important strategy for reducing the health care sector’s carbon footprint. Additionally, the findings enable quantification of telemedicine-associated CO_2_ emissions savings.

## Appendix

Multimedia Appendix 1 Comparison of doxy.me session characteristics to other national data (state, specialty).

Multimedia Appendix 2 Geographical distribution of CON2 emissions savings per session (N=79,904).

## Abbreviations

CMS: Centers for Medicare & Medicaid Services
CO_2_: carbon dioxide
EIO-LCA: economic input-output lifecycle assessment
GHG: greenhouse gas
HIPAA: Health Insurance Portability and Accountability Act
NPPES: National Plan and Provider Enumeration System
