# Supplementary material for: Travel Distance Between Participants in US Telemedicine Sessions With Estimates of Emissions Savings: Observational Study
Source: J Med Internet Res. 2024 May 15;26:e53437. doi: 10.2196/53437 (PMC11137427; doi:10.2196/53437)
Supplement: Multimedia Appendix 1 [file jmir_v26i1e53437_app1.docx]

**Multimedia Appendix 1.** Comparison of doxy.me session characteristics to other national data (state, specialty).

**Table S1.** Geographic distribution of telemedicine sessions, doxy.me* and Medicare**.

| **State (%)** | **Doxy.me**  **(N= 6,997,096)** | **Medicare**  **(N= 26,328,410)** | **Absolute Difference (%)** |
| --- | --- | --- | --- |
| **California** | 9.693951 | 11.04046162 | -1.346510615 |
| **New York** | 8.556177 | 7.955186052 | 0.600990948 |
| **Texas** | 6.253876 | 7.589964605 | -1.336088605 |
| **Massachusetts** | 5.454829 | 5.082012169 | 0.372816831 |
| **Florida** | 5.435492 | 7.78045465 | -2.34496265 |
| **Pennsylvania** | 5.188303 | 4.100243805 | 1.088059195 |
| **New Jersey** | 4.581536 | 4.250454167 | 0.331081833 |
| **Illinois** | 4.130377 | 4.112071333 | 0.018305667 |
| **Maryland** | 3.962879 | 3.374191605 | 0.588687395 |
| **Virginia** | 3.809129 | 2.646718887 | 1.162410113 |
| **Michigan** | 3.807657 | 3.192034764 | 0.615622236 |
| **North Carolina** | 3.406819 | 2.95835943 | 0.44845957 |
| **Washington** | 3.234118 | 1.682490511 | 1.551627489 |
| **Connecticut** | 2.893991 | 1.432596955 | 1.461394045 |
| **Ohio** | 2.729708 | 2.649677668 | 0.080030332 |
| **Georgia** | 2.444589 | 2.130793314 | 0.313795686 |
| **Minnesota** | 2.041193 | 1.705047893 | 0.336145107 |
| **Arizona** | 1.75889 | 2.134135711 | -0.375245711 |
| **Oregon** | 1.69482 | 0.925931342 | 0.768888658 |
| **Colorado** | 1.522448 | 1.256764081 | 0.265683919 |
| **Tennessee** | 1.327553 | 1.173925809 | 0.153627191 |
| **South Carolina** | 1.294739 | 1.373596051 | -0.078857051 |
| **Kentucky** | 1.041348 | 1.262047347 | -0.220699347 |
| **Missouri** | 1.038261 | 1.278231386 | -0.239970386 |
| **Indiana** | 0.977135 | 1.897566165 | -0.920431165 |
| **Wisconsin** | 0.945894 | 1.269670292 | -0.323776292 |
| **New Hampshire** | 0.900818 | 0.454820477 | 0.445997523 |
| **Rhode Island** | 0.809852 | 0.403909693 | 0.405942307 |
| **Maine** | 0.788471 | 0.560595949 | 0.227875051 |
| **Oklahoma** | 0.779453 | 1.189730029 | -0.410277029 |
| **Nevada** | 0.739322 | 0.839017624 | -0.099695624 |
| **Utah** | 0.636894 | 0.534434096 | 0.102459904 |
| **Alabama** | 0.594319 | 1.089074502 | -0.494755502 |
| **Iowa** | 0.57987 | 0.801259932 | -0.221389932 |
| **Vermont** | 0.515343 | 0.405960709 | 0.109382291 |
| **Arkansas** | 0.513642 | 0.887862199 | -0.374220199 |
| **Delaware** | 0.510484 | 0.698572379 | -0.188088379 |
| **Hawaii** | 0.491133 | 0.326001456 | 0.165131544 |
| **Idaho** | 0.450045 | 0.280271387 | 0.169773613 |
| **Kansas** | 0.414473 | 0.720115647 | -0.305642647 |
| **New Mexico** | 0.412114 | 0.585227137 | -0.173113137 |
| **Nebraska** | 0.377757 | 0.38183468 | -0.00407768 |
| **Louisiana** | 0.366253 | 1.217103502 | -0.850850502 |
| **West Virginia** | 0.269241 | 0.512826259 | -0.243585259 |
| **Montana** | 0.202113 | 0.226223308 | -0.024110308 |
| **Mississippi** | 0.127367 | 0.926896079 | -0.799529079 |
| **Alaska** | 0.121865 | 0.207710986 | -0.085845986 |
| **North Dakota** | 0.079433 | 0.141922737 | -0.062489737 |
| **South Dakota** | 0.06637 | 0.220206993 | -0.153836993 |
| **Wyoming** | 0.027654 | 0.133794635 | -0.106140635 |

*Dyadic telemedicine sessions of length 5-120 minutes, conducted using the doxy.me platform between Jan. 1, 2022 – Mar. 31, 2023.

**Medicare fee-for-service payments during the year 2020 as reported in Grace, S. (2020). Telehealth Expansion in Medicare: Policy Changes, Recent Trends in Adoption, and Future Impact. Retrieved from: https://carejourney.com/telehealth-expansion-in-medicare-policy-changes-recent-trends-in-adoption-and-future-impact/

**Table 2.** Specialty associated with telemedicine sessions, doxy.me* and Medicare**.

| **Specialty** | **Doxy.me (N= 6,997,096)** | **Medicare**  **(N= 26,328,410)** | **Absolute Difference (%)** |
| --- | --- | --- | --- |
| Missing Data | 56.44725654 | 14.02985075 | 42.41740579 |
| Mental Health Services | 22.47786725 | 5.485074627 | 16.99279263 |
| Social Worker | 8.209123193 | 8.097014925 | 0.112108268 |
| Psychiatry | 4.24648627 | 6.380597015 | -2.134110745 |
| Other Medical Specialty | 2.12574401 | 20.63432836 | -18.50858435 |
| Family Medicine | 2.008202871 | 12.6119403 | -10.60373743 |
| Nurse Practitioner | 1.412994548 | 12.68656716 | -11.27357262 |
| Internal Medicine | 1.291702096 | 16.30597015 | -15.01426805 |
| Nursing Services | 0.744010404 | 0 | 0.744010404 |
| Physician Assistant | 0.687740709 | 3.768656716 | -3.080916007 |
| Other (non-medical) | 0.348872105 | 0 | 0.348872105 |

*Dyadic telemedicine sessions of length 5-120 minutes, conducted using the doxy.me platform between Jan. 1, 2022 – Mar. 31, 2023. Specialty associated with each session by deterministic linkage of provider first name, last name, and approximate geographical location (zip code) with the CMS National Plan and Provider Enumeration System (NPPES).

**Medicare fee-for-service payments during the year 2020 as reported in Grace, S. (2020). Telehealth Expansion in Medicare: Policy Changes, Recent Trends in Adoption, and Future Impact. Retrieved from: https://carejourney.com/telehealth-expansion-in-medicare-policy-changes-recent-trends-in-adoption-and-future-impact/
