# Supplementary figures and images for: Travel Distance Between Participants in US Telemedicine Sessions With Estimates of Emissions Savings: Observational Study
Source: J Med Internet Res. 2024 May 15;26:e53437. doi: 10.2196/53437 (PMC11137427; doi:10.2196/53437)

**Multimedia Appendix 2.** Geographical distribution of CON2 emissions savings per session (N=79,904).

**
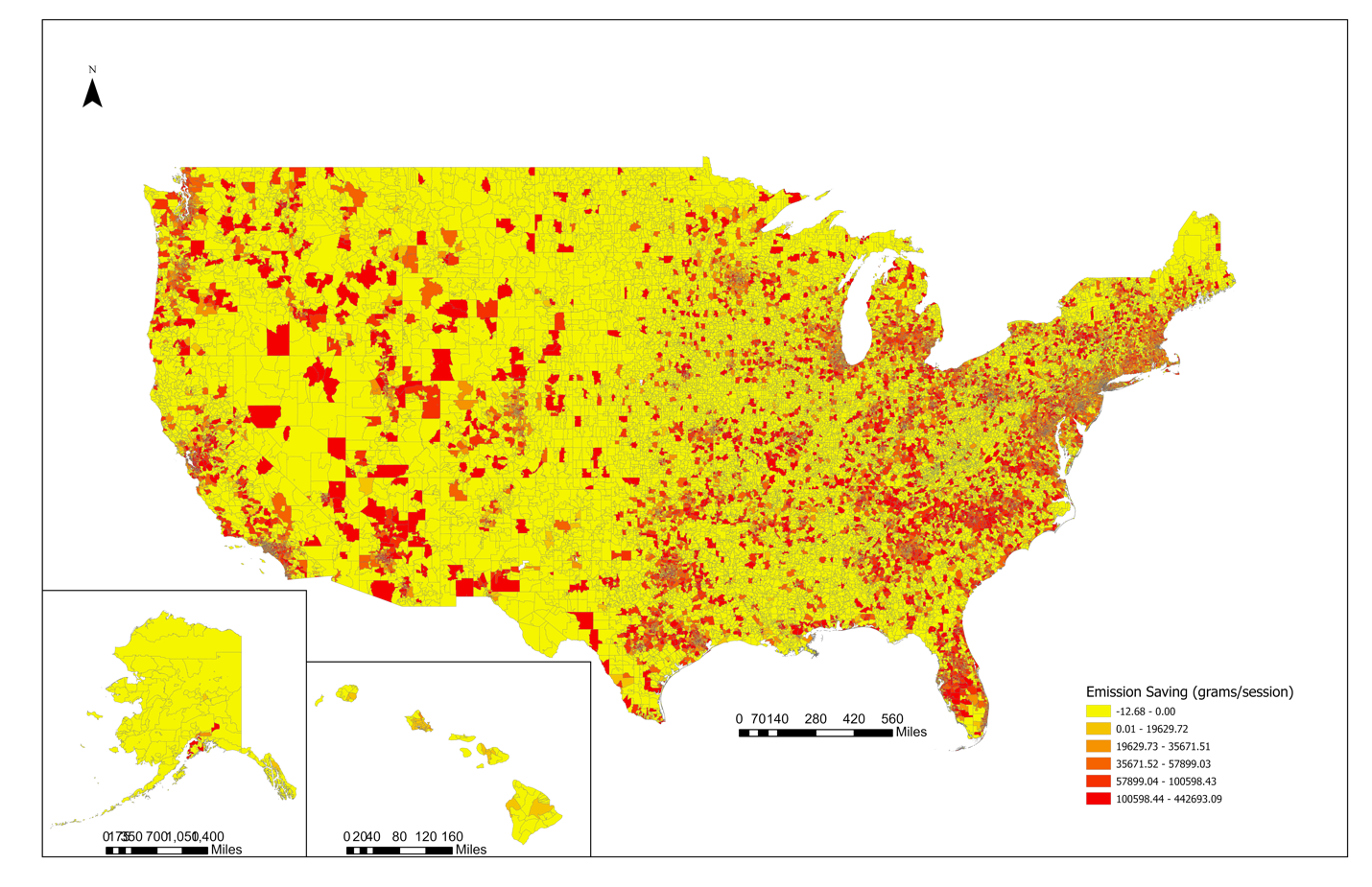
**

Supplement: Multimedia Appendix 2 [file jmir_v26i1e53437_app2.docx]
